# Increased isoprostane and prostaglandin are prominent in neurons in Alzheimer disease

**DOI:** 10.1186/1750-1326-2-2

**Published:** 2007-01-22

**Authors:** Gemma Casadesus, Mark A Smith, Samar Basu, Jing Hua, Dae E Capobianco, Sandra L Siedlak, Xiongwei Zhu, George Perry

**Affiliations:** 1 Department of Neuroscience, Case Western Reserve University, Cleveland, Ohio, USA; 2 Department of Pathology, Case Western Reserve University, Cleveland, Ohio, USA; 3 Faculty of Medicine, Uppsala University, Uppsala, Sweden; 4 College of Sciences, University of Texas at San Antonio, Texas, USA

## Abstract

**Background:**

Inflammation and oxidative stress are both involved in the pathogenesis of Alzheimer disease and have been shown to be reciprocally linked. One group of molecules that have been directly associated with inflammation and the production of free radicals are the prostaglandin 13,14-dihydro 15-keto PGF_2α _and the isoprostane 8-iso-PGF_2α_.

**Results:**

To further delineate the role of inflammatory and oxidative parameters in Alzheimer disease, in this study we evaluated the amount and localization of 13,14-dihydro 15-keto PGF_2α _and 8-iso-PGF_2α _in hippocampal post mortem tissue samples from age-matched Alzheimer disease and control patients. Our results demonstrate increased levels of 13,14-dihydro 15-keto PGF_2α _and 8-iso-PGF_2α _in the hippocampal pyramidal neurons of Alzheimer disease patients when compared to control patients.

**Conclusion:**

These data not only support the shared mechanistic involvement of free radical damage and inflammation in Alzheimer disease, but also indicate that multiple pathogenic "hits" are likely necessary for both the development and propagation of Alzheimer disease.

## Background

Alzheimer disease (AD) is the leading cause of senile dementia, with a prevalence that is directly related to age [1]. Over 4 million individuals are currently affected with the disease in the United States alone and this number is projected to increase to 14 million by 2050 [2]. At the present time, therapeutic management of the disease is primarily focused on palliative treatment of the symptoms rather than forestalling the progression of the disease [3] and the major obstacle in designing a rationale for therapeutic targets is our incomplete understanding of pathogenesis. To this end, it is imperative that the mechanistic hallmarks of this disease are established.

The tight association between aging and AD has led the field to propose oxidative stress as a major mechanism responsible for the onset and progression of AD [4]. Physiologically, the production of reactive oxygen species (ROS) is found in all aerobic organisms and arises from the secondary production of superoxide, hydrogen peroxide and the reaction of superoxide with nitric oxide (peroxynitrite) during metabolic and extra-metabolic processes of all cells. In AD, the excess formation of ROS is evident by signature reactions with critical biological molecules yielding damage to every category of biomacromolecules: sugars, lipids, proteins and nucleic acids [4]. That such oxidative damage occurs as one of the earliest aberrations in the disease indicates a major role of free radical damage in both etiology and pathogenesis.

In addition to direct oxidation damage of cellular macromolecules, free radical formation can also lead to damage indirectly by activating other harmful mechanisms such as inflammation [5]. In this regard, oxidative stress and inflammation are reciprocally linked such that inflammatory processes lead to increases in ROS production [6] and vice versa [7]. Given this interdependence, it is perhaps not surprising that ROS and inflammation can both be attenuated by individually targeted treatments, i.e., antioxidant or non-steroidal anti-inflammatory drug treatment [6,8,9].

Of note in this regard, epidemiological studies indicate a reduced risk of AD among users of anti-inflammatory drugs. Animal studies demonstrate that the capacity of non-steroidal anti-inflammatory drugs (NSAIDs) is to reduce the amount of plaque formation in mouse models of the disease. NSAIDs work mainly through the inhibition of cyclooxygenase, which is a critical component of the inflammatory response [10]. Therefore, anti-inflammatory drugs such as NSAIDs have become the focus of several new treatment strategies [11,12].

Like oxidative stress, inflammatory processes have been associated with AD and thought to play a major role in its onset and progression. In this regard, cytokines, such as interleukin-1 (IL-1), interleukin-6 (IL-6), tumor necrosis factor α (TNF-α) and transforming growth factor beta (TGF-β) are all affected and likely contribute to the inflammatory activation of microglia and astroglia [13].

One group of novel molecules that establish the link between inflammation and oxidative stress are prostaglandins and isoprostanes, respectively [14,15]. Prostaglandins are a group of 20-carbon containing hormone-like fatty acid derivatives that are produced by catalyzed cyclooxygenase of the arachidonic acid and localized to various tissues in the body [16]. Prostaglandins are important mediators of the inflammatory process [17,18] and 13,14-dihydro 15-keto PGF_2α _a major metabolite of prostaglandin F_2α _(PGF_2α_), is shown to be a potent indicator of *in vivo *cyclooxygenase (COX)-mediated inflammatory processes [19-22]. On the other hand, F_2_-isoprostanes, prostaglandin-like novel compounds are formed during free-radical catalyzed, non-enzymatic peroxidation of arachidonic acid [23] and, as such, considered to be reliable indicators of oxidative stress *in vivo *[14,22,24].

With regard to AD, some studies have shown increased F2-isoprostane levels in plasma or urine of AD patients compared to age-matched controls [25-27], however these findings remain controversial [28-30]. In addition, increased CSF levels of F_2_-isoprostanes have also been shown in AD patients [25,28,31-33], which, importantly, can be suppressed by antioxidant treatment [34]. While levels within the brain have been found to be increased in regions vulnerable to the disease [35,36], controversy remains as to localization (i.e., glia or disease-vulnerable neurons) and whether increased F_2_-isoprostane levels in AD are associated with the progression of the disease or rather are simply markers of increased gliosis [30]. In an attempt to clarify this controversy and accurately localize the presence of both PGF_2α _and F_2_-isoprostanes, we used an immunohistochemical method to examine both the levels and the subcellular localization of PGF_2α _and F_2_-isoprostanes in the AD brain.

## Results

**Figure 1:**
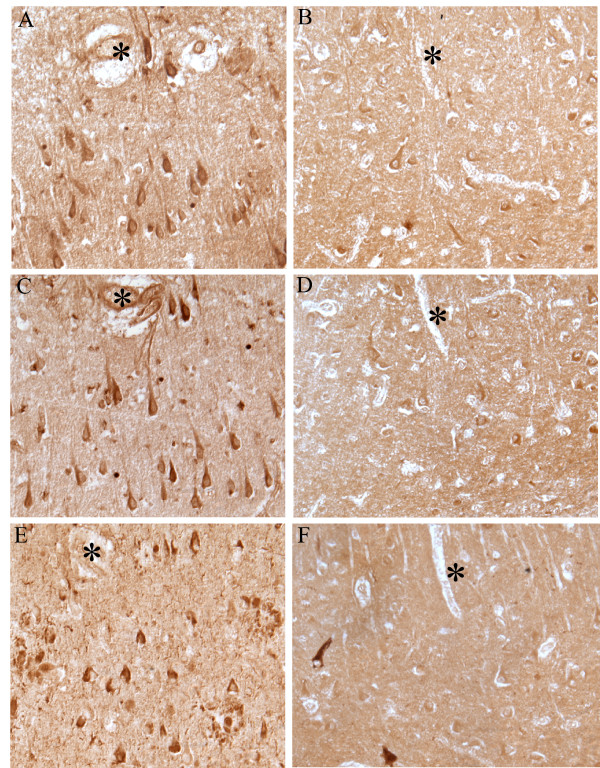
Isoprostanes localization in AD and control brain. In adjacent serial sections of hippocampus of AD cases, neurons are intensely labeled with antisera against 13,14-dihydro 15-keto PGF_2α _(A) and 8-iso-PGF_2α _(C). AT8 recognizes NFT in the same field (E). In adjacent serial sections from an age-matched control, neuronal levels of 13,14-dihydro 15-keto PGF_2α _(B) and 8-iso-PGF_2α _(D) are significantly lower. Only a few NFT recognized by AT8 are present in the control. (*) marks landmark vessels in series (A,C,E) and (B,D,F). Scale bar = 50 μm.

Detailed microscopic localization of 8-iso-PGF_2α _and 13, 14-dihydro 15-keto PGF_2α _in the AD cases revealed strong labeling of the cytoplasm of neurons, when compared to age-matched control cases (Figure 1). Neuronal populations principally affected were large pyramidal neurons of the hippocampal formation (CA-1, CA-2, and CA-3/4), subiculum, pre-α layer of the entorhinal cortex, and cerebral neocortex. While neurons affected by neurofibrillary pathology showed labeling with both antibodies, there was no predilection for pathologically altered neurons, as the majority of the labeling occurred in the perikaryal cytoplasm of morphologically normal pyramidal neurons. Dystrophic neurites and neuropil threads were not observed with 13,14-dihydro 15-keto PGF_2α _and 8-iso-PGF_2α _antibodies. No labeling of neuritic plaques or parenchymal amyloid-β deposits could be discerned. In addition to neuronal immunoreactivity, immunolabeling of reactive astrocytes, generally in parallel with the perinuclear accumulations of glial filaments, was noted diffusely in some sections. Blood vessels, ependymal cells, and choroid plexus epithelium showed no significant immunoreactivity. Demonstrating the specificities of our findings, antibodies directed against porcine-thyroglobulin linked 13,14-dihydro 15-keto PGF_2α _and 8-iso-PGF_2α _revealed very similar staining patterns of increased neuronal as well as glial cells in AD cases. Moreover, omission of the primary antibodies completely abolished immunoreactivity (data not shown).

**Figure 2:**
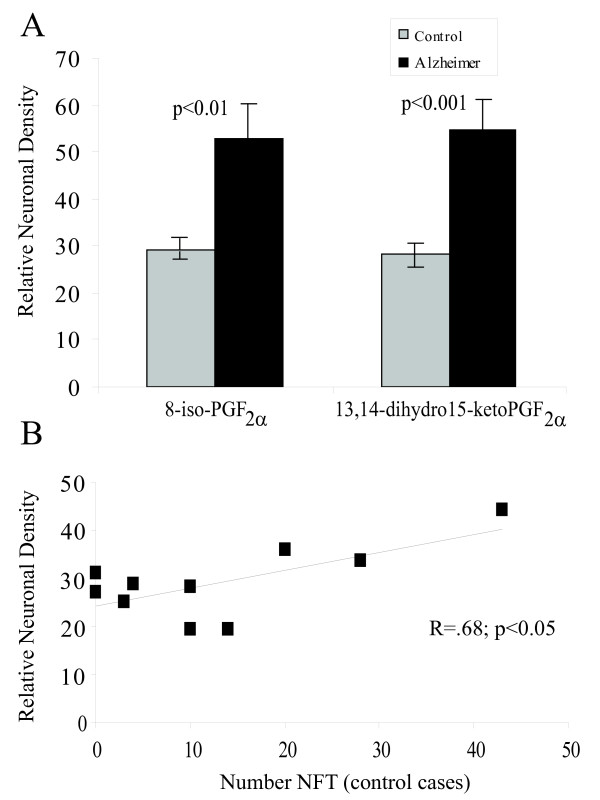
Quantification of neuronal levels of isoprostanes. A. Computer assisted image analysis reveals pyramidal neurons from cases of AD (n = 10) reveal significantly higher levels of 8-iso-PGF_2α _(p < 0.01) and13,14-dihydro 15-keto PGF_2α _(p < 0.001) than aged controls (n = 10). Mann-Whitney U-test. Data shown expresses mean +/- SEM. B. In the aged control cases, neuronal levels of 8-iso-PGF_2α _are significantly correlated with the numbers of AT8-positive NFT.

Quantification of the relative densities of pyramidal neurons stained revealed a statistically significant increase in the immunoreactivities for 8-iso-PGF_2α _(p < 0.01) and PGF_2α _metabolite (p < 0.001) in AD cases (Figure 2A). In the same three fields on adjacent serial sections stained for phosphorylated tau, the number of immunostained neurofibrillary tangles (NFT) was quantified. In the AD cases, the number of NFT within the three fields analyzed ranged from 31–293 NFT, mean of 121. Eight of the 10 age-matched control cases contained small numbers of AT8-positive NFT, ranging from 3–43 in the fields analyzed, mean of 20.3. Relative neuronal density of 8-iso-PGF_2α _or PGF_2α _metabolite in the AD cases showed no correlation with the number of NFT (r = 0.12), while the control cases showed a significant positive correlation (r = .68; p < 0.05) of NFT numbers with 8-iso-PGF_2α _(Fig 2B). No significant correlation was noted between levels of 13,14-dihydro 15-keto PGF_2α _and 8-iso-PGF_2α _with age in either the control or AD cases.

## Discussion

The data presented in this study shows both PGF_2α _and F_2_-isoprostanes are increased in hippocampal tissue collected from AD patients compared to non-AD patients, indicating that brain inflammation and oxidative stress are significantly higher in AD compared to the aged-matched controls. This study is the first of its kind to report an increased level of a prostaglandin F_2α_-metabolite, which corresponds to the level of the COX-mediated primary prostaglandin F_2α_, in the brains of individuals affected by AD. This data certainly augments the work of Ho et al, who carefully characterized the appearance and progression of neuronal accumulation of COX-2 as both a function of clinical course of the disease as well as within different neuronal populations of the hippocampus [37]. Their findings highlight the COX-2 involvement early in the disease course which provides a reasonable and prudent target for therapeutics such as NSAIDS. This is extremely relevant to the current work on prostaglandins, whose appearance coincides with the cellular location and development of pathology as seen with COX-2.

Prostaglandins are well-known mediators of inflammation [17]. 15-Keto-dihydro-PGF_2α_, a metabolite of bioactive PGF_2α _metabolized through 13,14-dihydro 15-keto PGF_2α _dehydrogenases in most of the tissues in the body is a potent indicator of in vivo inflammatory processes [19-22]. Thus, the results from this study suggest a local ongoing COX-related inflammatory process among patients with AD, which possibly plays a major role in the onset or progression of the disease. In addition, PGF_2α _is shown to be a potent vasoconstrictive compound [38] which also may play a role in the progression of AD in which vascular degeneration is a characteristic [4]. Notably, amyloid-β itself can act as a pro-inflammatory agent causing the activation of many of the inflammatory components, including glial activation [39,40] and, in somewhat of a feedforward manner, cyclooxygenases potentiate the generation of amyloid-β [41]. However, it is important to note that our findings revealed little correlation between amyloid and 13,14-dihydro 15-keto PGF_2α _and 8-iso-PGF_2α_. Similarly, while no correlation was found with phosphorylated tau among the AD cases, a positive correlation was seen among the controls, indicating the inflammatory response as an early change. Clinical data on the mental status of the control cases used in this study was not available, therefore further analysis using clinically followed cases of mild cognitive impairment would be required to faithfully answer this question. These findings are not only consistent with findings of other oxidative adducts [42,43], but also emphasize the often different properties of amyloid-β and phosphorylated tau *in vivo *compared to *in vitro *[44,45].

Another novel finding in this study is that the levels of F_2_-isoprostanes are increased in hippocampal sections from patients with AD as compared to the control subjects. These findings are in accordance with previous mass spectrometry studies demonstrating increased levels of F_2_-isoprostanes in the AD brain [46]. Our study has now shown the specific localization of these proteins in the AD brain demonstrating that these adducts strikingly localize to vulnerable neurons in the disease as well as other cellular types like glia, often associated with inflammatory responses. F_2_-isoprostanes are now regarded as one of the most reliable indicators of oxidative stress *in vivo *[15,47]. Elevated level of isoprostanes has been shown in CSF and brain tissue previously [35,48], however, there are controversies regarding the levels of isoprostanes in the plasma or urine [15]. One study describes higher levels of isoprostanes in the plasma and urine [25], while others do not [28,29] The accumulation of both PGF_2α _and F_2_-isoprostanes in cerebral tissues collected from AD are an unique finding since both of these compounds possess extremely short half-lives [16,49,50]. In this regard, elevations of these compounds are seen only for a short period of time in experimental acute inflammation protocols (e.g., septic shock) following cardiac arrest or cardio pulmonary bypass surgery, which rapidly decrease to the initial basal levels [19,21,51]. Nevertheless, a more persistent rise of basal levels of these compounds is observed during chronic inflammation such as in various rheumatic diseases and type 2 diabetes [15]. Therefore, as previously suggested [52], the levels of oxidative stress are likely low and chronic in nature.

Notably, and in addition to being excellent markers of inflammation, both PGF_2α _and F_2_-isoprostanes (mainly 8-iso-PGF_2α_) possess independent bioactive properties. That no age related increase was found in either the normal or disease tissue suggests a more direct role in disease pathogenesis. In this regard, PGF_2α _and F_2_-isoprostanes (mainly 8-iso-PGF_2α_) are potent vasoconstrictive agents, and PGF_2α _is a well known mediator of pain and inflammation [17]. Further it has recently been shown that 8-iso-PGF_2α _can induce PGF_2α _release and thereby inflammatory responses in rabbits [15]. Therefore, the accumulation of PGF_2α _and F_2_-isoprostanes in the cerebral tissues not only indicates the presence of massive inflammation in the AD brain but also highlights the impact that these compounds could have independent bioactive entities, where a chronic accumulation of these compounds in the brain could further worsen the status of inflammation and oxidative stress and thus the pathophysiology of the disease. These latter findings together with the current findings of *in situ *localization of F_2_-isoprostanes in the hippocampal or cortical tissue samples from AD patients further supports the notion that locally involved oxidative stress together with the inflammatory response is possibly one of the major mechanistic hallmarks of AD and therefore represent therapeutic intervention points of great potential. However, despite the apparent effectiveness for NSAIDs, which are COX inhibitors, and antioxidants on preventing the risk of developing AD consistently reported in epidemiology studies [53,54], clinical trials with these drugs demonstrate little to no effect in AD patients [55]. These findings highlight the importance of early interruption of pathogenic processes, consistent with our finding of a positive correlation between neuronal 8-iso-PGF_2α _and NFT numbers in control cases but not in AD cases.

In conclusion, this study presents an accumulation of both prostaglandins and isoprostanes in the brain tissues of patients with AD which further advocates for importance of inflammation and oxidative stress in this disease and treatment strategies that counteract inflammatory processes and oxidative stress simultaneously.

## Methods

### Tissue

Hippocampal and cortical tissue samples were obtained post mortem from patients with histopathologically confirmed AD (n = 21, ages 61–96 years, mean = 80.8 years). Control cases used in this study included 3 young (ages 17, 23, and 43 years) and 13 aged-matched controls (ages 60–91 years, mean 72.9 years) with similar post mortem intervals (AD: 3–37 hr, mean 14.3 hour; controls: 3–48 hr, mean 19.1 hr). All cases were categorized based on clinical and pathological criteria established by CERAD and NIA consensus panel [56,57]. From the clinical reports available to us, we found no obvious differences in agonal status or other potential confounders between the groups. Tissue was fixed in methacarn (methanol: chloroform: acetic acid; 6: 3: 1 v/v/v) at 4°C overnight. Following fixation, tissue was dehydrated through ascending ethanol, embedded in paraffin, and 6-μm sections were placed on silane-coated slides (Sigma, St. Louis, MO, USA).

### Immunocytochemistry

Tissue sections were de-paraffinized in xylene, hydrated through descending ethanol, and endogenous peroxidase activity quenched by a 30 min incubation in 3% hydrogen peroxide in methanol. Non-specific binding sites were blocked by a 30 min incubation in 10% normal goat serum. Tissue sections were immunostained using the peroxidase/anti-peroxidase method with 3-3'-diaminobenzidine as co-substrate as previously described [5]. Antibodies used were: 1) rabbit polyclonal antibody to free 13,14-dihydro 15-keto PGF_2α _[22]; 2) rabbit polyclonal antibody to free 8-iso-PGF_2α _[58]; 3) rabbit polyclonal antibody to free 8-iso-PGF_2α _conjugated to porcine thyroglobulin (Assay Designs, MI); and 4) rabbit polyclonal antibody to free 13, 14-dihydro 15-keto PGF_2α _(Cayman Chemical, MI). Sections were also immunostained with a monoclonal mouse antibody AT8, which recognizes phosphorylated tau (Ser202/Thr205) (Pierce, Rockford, IL) to identify the location of pathological structures. Control experiments included omission of primary antisera.

### Quantification

Quantification of 13, 14-dihydro 15-keto PGF_2α _and 8-iso-PGF_2α _protein immunoreactivity was performed as previously described [43]. The cases used included 10 AD (ages 65–87 years) and 10 age-matched controls (ages 67–82 years) that were randomly selected and stained with the same antisera [22,58] at the same time. Digital images were taken with an Axiocam camera (KS300, Zeiss, Munchen-Hallbergmoss, Germany) and compatible quantification software (Axiovision, Carl Zeiss Vision GmbH, Munchen-Hallbergmoss, Germany) used to determine the mean staining intensity of pyramidal neurons in the CA1/CA2 region of the hippocampus. Briefly, using the 20× objective, 3 fields were selected and the software outlined the immunostained structures and measured the intensity. The intensity of all unstained areas within each fields are determined as the background levels. In those cases where neuronal staining was at the background level and the software unable to delineate the cell bodies, all pyramidal neurons within each field, where nuclei were visible were outlined manually and measured as above. Mean neuronal intensities for each case were determined and because the n numbers were relatively small, the Mann-Whitney test applied (SigmaStat).

In the same fields on adjacent sections immunostained with monoclonal anti-phosphorylated tau (AT8), the numbers of NFTs were counted. Correlations were determined between the mean level of neuronal prostaglandin and isoprostane with the number of NFTs in the cases.

## Competing interests

The author(s) declare that they have no competing interests.

## Authors' contributions

GC, JH, DC, SLS collected the data. GC participated in the design of the study and performed the statistical analysis. MAS, XZ, SB, and GP conceived of the study, and participated in its design and coordination and helped to draft the manuscript. All authors read and approved the final manuscript.
